# Sampling data of macro-invertebrates collected in grasslands under restoration succession in a lowland stream-valley system

**DOI:** 10.3897/BDJ.12.e125462

**Published:** 2024-07-23

**Authors:** Gijs M. Gerrits, Lia Hemerik

**Affiliations:** 1 Wageningen University, Biometris, Department of Mathematical and Statistical Methods, Wageningen, Netherlands Wageningen University, Biometris, Department of Mathematical and Statistical Methods Wageningen Netherlands; 2 Netherlands Institute of Ecology (NIOO-KNAW), Department of Terrestrial Ecology, Wageningen, Netherlands Netherlands Institute of Ecology (NIOO-KNAW), Department of Terrestrial Ecology Wageningen Netherlands; 3 Naturalis Biodiversity Center, Evolutionary Ecology Group, Leiden, Netherlands Naturalis Biodiversity Center, Evolutionary Ecology Group Leiden Netherlands

**Keywords:** macro-arthropod survey, FAIR, Drentsche Aa, Anlooer Diepje, pyramid traps, pitfalls, Insecta, Araneae, Carabidae

## Abstract

**Background:**

Publication of data from past field studies on invertebrate populations is of high importance, as there is much added value for them to be used as baselines to study spatiotemporal population and community dynamics in these groups. Therefore, a dataset consisting of occurrence data on epigaeic invertebrates collected in 1996 was standardised into the Darwin core format and cross-checked in order to make it publicly available following FAIR data principles. With publication, it can contribute to the biodiversity assessment of terrestrial invertebrates, thereby improving the availability and accessibility of much-needed historical datasets on macro-invertebrates.

Here, we present sampling event data on invertebrates from four grasslands taken out of agricultural production over the span of several decades, effectively displaying a chronosequence on the effects of agricultural extensification. The data were collected by means of a standardised sampling design using pyramid traps, pitfall traps and soil samples.

**New information:**

The raw data presented in this data paper have not been published before. They consist of 20,000+ records of nearly 70,000 specimens from 121 taxonomic groups. The data were collected using a standardised field study set-up and specimens were identified by taxonomic specialists. Most groups were identified up to family level, with eight groups identified up to species level. The occurrence data are complemented by information on plant composition, meteorological data and soil physical characteristics. The dataset has been registered in the Global Biodiversity Information Facility (GBIF): http://doi.org/10.15468/7n499e

## Introduction

In 1996, a nine-month sampling programme was carried out in four semi-natural grasslands in the northern part of the Netherlands with the aim of studying the effects of restoration management in former agricultural grasslands on the composition of below- and above-ground macro-invertebrate communities (Hemerik and Brussaard 2002). The four grasslands had been taken out of agricultural production after a period of intensive land use with high levels of nutrient amendments. At the time of sampling, the grasslands formed a restoration succession chronosequence of 7, 11, 24 and 29 years post-agricultural management. The main aim of this extensification programme was to restore former botanical richness by reducing nutrient levels in the soil (Berg and Hemerik 2004). The 1996 field sampling programme was set up in order to study the effects of restoration management on soil and ground-dwelling insects and spiders. It was suspected that changes in plant biomass production and alterations in litter quantity and quality stemming from the cessation of nutrient amendments would affect macro-invertebrate populations (Hemerik and Brussaard 2002). In addition, changes in vegetation structure, affecting microclimate, might have consequences for soil and ground-dwelling animal groups. Therefore, the researchers wanted to explore whether they could find differences in species diversity and composition of macro-invertebrates in the chronosequence.

The sampling plots were situated in the valley of the brooklet "Anlooer" Diepje, a much intact natural stream that forms part of the stream-valley system "Drentsche Aa," which is situated in the north-eastern part of the Netherlands in the Province of Drenthe (Liu et al. 2021). The fields surrounding the brooklet mainly consist of grasslands on loamy sand with a slightly acidic pH. The field study set-up involved the use of photoeklektors, also known as pyramid emergence traps, to sample airborne insects emerging as imago from the soil. Inside the tent of the pyramid emergence trap, two pitfall traps were dug into the ground. The soil that was dug out for placing the pitfalls was taken to the laboratory as soil samples to hand-sort for macro-invertebrates. The pyramid and pitfall traps were emptied weekly and moved randomly to a new location within the experimental plot every two weeks. Concurrently, a number of environmental variables were collected, including soil physical characteristics, soil temperature, as well as plant composition in the experimental plots (see Table 3 for a complete list). The sampling programme ran for 9 months, from 11 March until 23 December 1996.

This resulted in a dataset containing 21,282 records of 71,415 specimens that were caught during the nine months of sampling. All invertebrate species were identified up to at least the taxonomic level of order. In total, 121 taxonomic groups were distinguished (see Table 3). Terrestrial isopods (Isopoda), millipedes (Diplopoda), centipedes (Chilopoda), spiders (Araneae), harvestmen (Opiliones), click beetles (Elateridae), ground beetles (Carabidae) and weevils (Curculionidae/Brentidae) were identified up to species level.

In order to make it publicly accessible and (re)usable following FAIR data objectives, the data have been reviewed and cross-checked, as well as reorganised and standardised following the Darwin Core Standard (Wieczorek et al. 2012). The data have subsequently been published on GBIF (Hemerik and Creuwels 2023): https://doi.org/10.15468/7n499e

## Project description

### Title

Macro-invertebrate survey Drentsche Aa 1996

### Personnel

The original monitoring in 1996 was performed by Lia Hemerik (ORCID 0000-0002-6892-2840), together with taxonomic specialists who assisted with field work and identified specific species groups. The specialists were Arnold Spee and Theo van Dijk at Biologisch Station Wijster, Matty Berg (ORCID 0000-0001-8442-8503), Aart Noordam, Michael Traugeot and Theodoor Heijerman (ORCID 0000-0002-9835-600X). Standardising the dataset into Darwin Core format, cross-checking and publication of the data were performed by Lia Hemerik, assisted by Gijs M. Gerrits (ORCID 000-0002-2315-9677).

### Study area description

**Figure 1.**
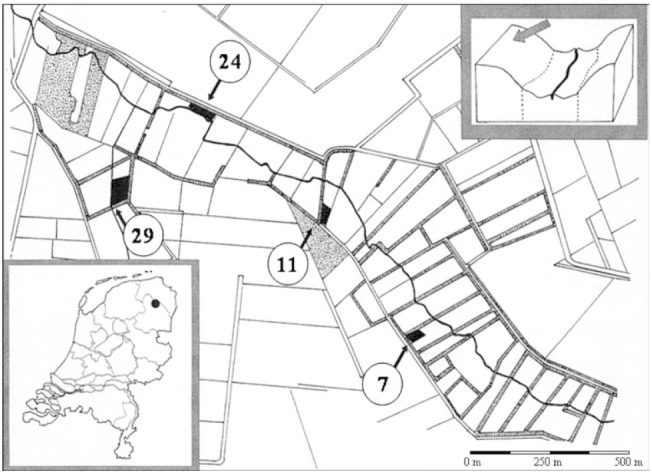
Location of the four grasslands in the valley of Anlooërdiepje in the north-eastern part of the Netherlands (see inset below left). The four black arrows point at the four experimental plots, indicated in black. The numbers in circles indicate the number of years the grassland was taken out of agricultural production at the time of sampling in 1996: plot O (7 years since last fertilisation), plot B (11 years since last fertilisation), plot C (24 years since last fertilisation) and plot K (29 years since last fertilisation). On each grassland, a randomly selected rectangle of 30 m by 15 m served as the sampling plot. See Suppl. material 1 for more information on palnt composition.

The study area is located in the Drentsche Aa stream valley in the Province of Drenthe in the north-eastern part of the Netherlands (Fig. 1). The Drentse Aa is a 30 km^2^ stream valley that has had a long history of agricultural exploitation with high levels of nutrient amendments and large alterations in the hydrological functioning of the Drentsche Aa stream and its tributaries, amongst which the Anlooer Diepje brooklet (Liu et al. 2021).

The four field study plots, all located along the banks of the Anlooer Diepje (Fig. 1), consisted of grass dominated vegetation, with *Loliumperenne* L., *Holcuslanatus* L., *Festucarubra* L. and *Anthoxanthumodoratum* L., respectively, dominating in the chronosequence gradient. Management consisted of mowing once a year and the resulting hay was removed (Verschoor et al. 2001).

### Funding

The reorganisation and publication of the data were made possible with a grant from NLBIF Netherlands Biodiversity Information Facility (grant number nlbif2021.003).

## Sampling methods

### Study extent

The sampling programme was designed as a block field study set up within randomly selected experimental plots that were situated in the four selected grasslands of the chronosequence.

### Sampling description

Within each of the four grasslands taken out of agricultural production, a plot measuring 30 m × 15 m was selected for the sampling to take place. Each plot was divided into three subplots (10 m × 15 m). One pyramid trap was placed within each of the subplots (i.e. 3 pyramid traps per experimental plot) and moved randomly across the subplot every two weeks. Inside the pyramid trap, two pitfall traps were placed (i.e. 6 pitfall traps per experimental plot), flush with the soil surface. The soil samples that were dug out (i.e. 12 soil samples per experimental plot) to accommodate the pitfall traps were collected in plastic bags and taken to the laboratory in Wageningen, stored at a temperature of 4°C and hand-sorted for soil macrofauna. Both pyramid and pitfall catches were collected on a weekly basis and soil samples on a two-weekly basis. In total, 12 pyramid traps were placed in the four plots, together with 24 pitfalls and 48 soil samples. See Table 1 for details.

**Figure 2.**
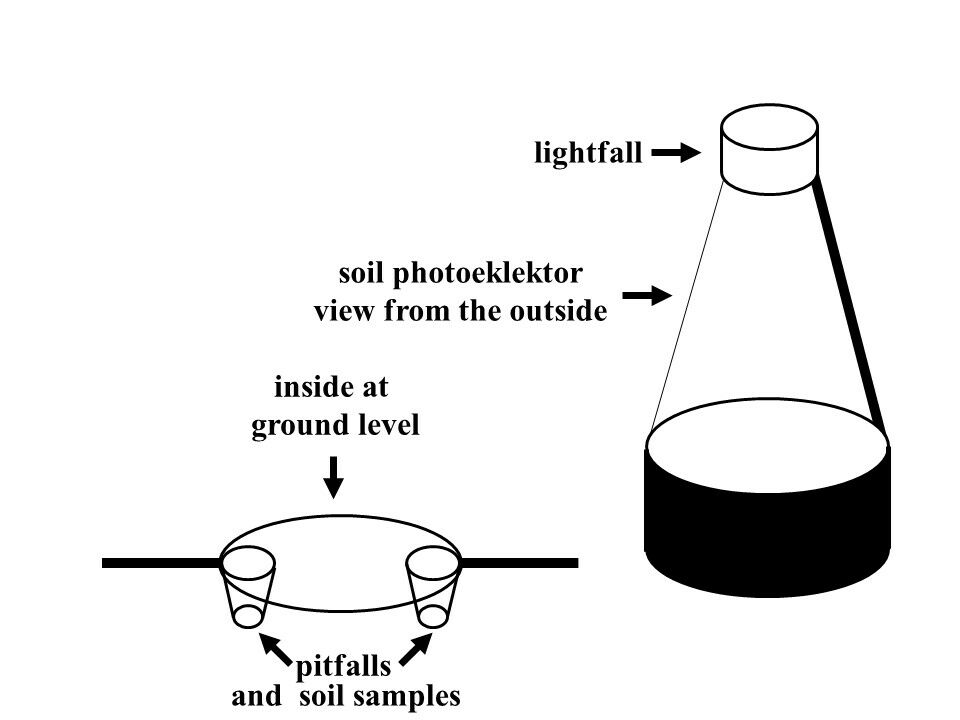
Sampling was set up with pyramid traps and pitfall traps. The pyramid trap was 80 cm high and had a diameter of 56 cm. Light traps and pitfalls were emptied weekly. Soil samples were taken every two weeks and, at the same time, the light traps and pitfalls were moved to a new location within the experimental plot.

The pyramid traps consisted of an opaque grey plastic ring (diameter of 56 cm). Above this ring, a gauze net made of white net fabric formed an inverted funnel-shaped dome. On top of the funnel dome, a white plastic trapping container in the shape of a ring functioned as a lightfall, bringing the total height of the pyramid trap to 80 cm (Fig. 2). The lightfall at the top contained a 2% formaldehyde (CH_2_O) solution, which served as a killing preservative. The pyramid traps were designed to catch flying insects that were either residing in the grass tussocks at the moment of placement or, more specifically, all insects that hatched from the soil during the time the trap was placed at each location. The pitfall traps consisted of white plastic cups (0.5-litre yoghurt cups) with a diameter of 9 cm. The pitfalls were also filled with a 2% formaldehyde solution.

The specimens that were collected during sampling were stored in 70% ethanol, awaiting identification. The samples were destroyed after identification.

Table 1 provides an overview of the four plots and the trap numbers that were placed in each sampling plot.

Additional data were collected during the sampling programme on abiotic and botanical variables (see Table 2 for an overview). In addition, photographs of the research plots taken in 2021 are provided in Suppl. material 1, together with an overview of botanical composition in 1996 and in 2021.

Soil wet weight and dry weight were measured from the soil cores collected for placement of the pitfall traps. Furthermore, mean air temperature and total weekly precipitation in the week preceding a collection event were calculated from data available from the KNMI Meteorological Station Eelde (KNMI 2023), which is situated approximately 11 km from the research site. The measurements are connected to the eventIDs and published as a MeasurementOrFact table.

As part of a study on nematodes in the same plots, Verschoor et al. (2001) collected abiotic data, namely bulk density, clay-silt percentage, pH-H_2_O, C-pool, N-pool and C/N ratio (see their table 1).

## Geographic coverage

### Description

The study area was located in the Province of Drenthe, in the north-eastern part of the Netherlands, see Fig. 1. The plots O, B, C and K have their own pair of coordinates in GBIF. For the location of these plots on the map, we refer to Fig. 1.

### Coordinates

53.0449 and 53.0502 Latitude; 6.6661 and 6.6799 Longitude.

## Taxonomic coverage

### Description

After collection, the specimens were identified by taxonomy specialists. All specimens were first sorted and identified to order level; true flies (Diptera) and beetles (Coleoptera) were further identified to family level by Lia Hemerik. Arnold Spee and Theo van Dijk assisted with field sampling and identification of the carabid beetles. Matty Berg identified terrestrial isopods (Isopoda), millipedes (Diplopoda) and centipedes (Chilopoda). Aart Noordam identified spiders (Araneae) and harvestmen (Opiliones). Michael Traugeot identified beetle larvae. Theodoor Heijerman identified weevils (Curculionidae/Brentidae).

Before publication of the dataset, all original taxonomical assignments were cross-checked with the Checklist Dutch Species Register – Nederlands Soortenregister (Creuwels and Pieterse 2023) and provided with a currently valid assignment when necessary. For a complete list of taxa included in the dataset, see Table 3.

## Temporal coverage

**Data range:** 1996-3-11 – 1996-12-23.

### Notes

The field study started on 11 March 1996 and was finished on 23 December 1996. Sampling points were created each week when traps were collected.

## Usage licence

### Usage licence

Other

### IP rights notes

CC-BY-NC 4.0

## Data resources

### Data package title

Macro-invertebrate survey Drentsche Aa 1996

### Resource link

https://www.gbif.org/dataset/a90782f8-e60e-46d8-82a4-e749b5cf69c3

### Alternative identifiers

https://ipt.nlbif.nl/resource?r=drentse_a; https://doi.org/10.15468/7n499e

### Number of data sets

1

### Data set 1.

#### Data set name

sampling event, 9 months in 1996, in the Drentsche A

#### Data format

txt

#### Description

The dataset has been published in the Global Biodiversity Information Facility platform, GBIF (Hemerik and Creuwels 2023). It is set up as a sampling event dataset with a three part structure; eventID, occurrenceID and MeasurementorFact. The dataset is published as a Darwin Core Archive (DwCA). The core data file contains 2,898 events with 21,887 occurrences. The GBIF IPT (Integrated Publishing Toolkit, Version 2.5.6) serves as the data repository. The table below provides descriptions of the column labels used. Note that labels are entered in alphabetical order, not in the order they are provided in the DwCA; MoF stands for Measurement-or-Fact extension.

**Data set 1.** 

Column label	Column description
basisOfRecord (Occurrence extension)	the specific nature of the data record.
class (Occurrence extension)	class name.
coordinateUncertaintyInMetres (Event core)	coordinate uncertainty.
country (Event core)	the country where the samples were taken, i.e. the Netherlands.
countryCode (Event core)	the code of the country where the samples were taken, i.e. NL.
decimalLatitude (Event core)	latitude of the field in which the sampling was performed.
decimalLongitude (Event core)	longitude of the field in which the sampling was performed.
endDayOfYear (Event core)	end of sampling period.
eventDate (Event core)	date of emptying the pitfall or lightfall or of taking a soil sample.
eventID (Event core, Occurrence extension, MoF)	unique identifier for each event per date, per pitfall, lightfall or soil sample.
eventRemarks (Event core)	remarks of emptying the pitfall or lightfall or of taking the soil sample.
family (Occurrence extension)	family name.
geodeticDatum (Event core)	the ellipsoid, geodetic datum or spatial reference system (SRS), upon which the geographic coordinates given in decimalLatitude and decimalLongitude are based.
identifiedBy (Occurrence extension)	a list of names of people who assigned the taxon to the subject.
individualCount (Occurrence extension)	number of recorded specimens per occurrenceID.
kingdom (Occurrence extension)	kingdom name.
lifestage (Occurrence extension)	life stage at which specimen was caught.
locality (Event core)	Drentsche Aa, one of the four fields.
locationRemarks(Event core)	different descriptions of the samples.
measurementAccuracy (MoF)	the accuracy of the measurement.
measurementDeterminedBy (MoF)	institution or person who has performed the measurement.
measurementRemarks (MoF)	comments on how the value is obtained.
measurementType (MoF)	description of the measurement, for example, minimum temperature in the week before, total precipitaion in the week before.
measurementUnit (MoF)	the unit of the measurement in SI units, e.g. g, mm.
measurementValue (MoF)	the measured/observed value.
occurrenceID (Occurrence extension)	unique identifier for each occurrence per species, per date, per pitfall, lightfall or soil sample.
occurrenceStatus (Occurrence extension)	a statement about the presence or absence, here present.
order (Occurrence extension)	order name.
ownerInstitutionCode (Event core)	the name (or acronym) in use by the institution having ownership of the object(s) or information referred to in the record.
parentEventID (Event core)	groups the eventIDs of 1 lightfall plus 2 pitfalls or of upper and lower soil samples.
phylum (Occurrence extension)	phylum name.
recordedBy (Occurrence extension)	institution by which specimen is identified and recorded.
sampleSizeUnit (Event core)	g resp. number.
sampleSizeValue (Event core)	size of soil sample, cumulation of individuals.
samplingEffort (Event core)	the amount of effort expended during sampling.
samplingProtocol (Event core)	the names and descriptions of the methods or protocols used during sampling.
scientificName (Occurrence extension)	genus + specificEpithet + author + publication year.
sex (Occurrence extension)	the sex of the biological individual.
startDayOfYear (Event core)	start of sampling period.
subfamily (Occurrence extension)	subfamily name.
taxonRank (Occurrence extension)	the taxonomic rank of the most specific name in the scientificName.
taxonRemarks (Occurrence extension)	Comments or notes about the taxon or name.
type (Event core)	event.
verbatimIdentification (Occurrence extension)	the taxonomic identification as it appeared in the original record.

## Supplementary Material

BACE19D0-B8EF-5746-B042-AC09956CA88D 10.3897/BDJ.12.e125462.suppl1 Supplementary material 1 Supplementary material Drentsche Aa 1996 Data type word file Brief description Vegetation composition in 1996 and photographic overview of current vegetation (2021). File: oo_1042587.docx https://binary.pensoft.net/file/1042587 Lia Hemerik & Gijs M. Gerrits

## Figures and Tables

**Table 1.** Details on sampling plots (named K, O, C and B), years since extensification (YSE) and trap and soil sample numbers per plot. Note that soil samples are divided into up-soil (top 10 cm) and low-soil (10–20 cm deep) samples, but numbers and plots are the same for both types.

Plot	YSE	Pyramid trap (lf)	Pitfall (pf)	Soil cores (up_soil & low_soil)
O	7	lf4–lf6	pf7–pf12	_soil7– _soil12
B	11	lf10–lf12	pf19–pf24	_soil19–_soil24
C	24	lf7–lf9	pf13–pf18	_soil13–_soil18
K	29	lf1–lf3	pf1–pf6	_soil1–_soil6

**Table 2.** Environmental variables (a total of 10) can be found in the GBIF Dataset "Macro-invertebrate survey Drentsche Aa 1996". The plant composition is in Suppl. material 1.

**Variable**	**Unit**
Wet weight of upper 7.5 cm of the soil core (WWu)	g
Wet weight of the soil core 7.5–15 cm deep WWl)	g
Dry weight soil of upper 7.5 cm of the soil core (DWu)	g
Dry weight soil of the soil core 7.5–15 cm deep (DWl)	g
Moisture content soil the soil core 0–7.5 cm deep (= 100 * (WWu-DWu)/WWu)	%
Moisture content soil the soil core 7.5–15 cm deep (= 100 * (WWl-DWl)/WWl)	%
Mean precipitation	mm
Maximum air temperature	°C
Minimum air temperature	°C
Mean air temperature	°C
Plant cover (Braun-Blanquet) per species	cover %

**Table 3.** Taxonomic list of all species caught during the 1996 sampling programme. The list is organised alphabetically from highest to lowest taxonomic level; first by phylum, then class, order and finally by taxon. Note that taxon contains three levels of identification; subfamily, genus and species, which are in this sequence dealt with within the groups.

**Group**	**Phylum**	**Class**	**Order**	**Family**	**Taxon**	**n**
earthworms	Annelida	Oligochaeta	Opisthopora	Lumbricidae		258
arachnids	Arthropoda	Arachnida	Araneae	Linyphiidae		60
arachnids	Arthropoda	Arachnida	Araneae	Linyphiidae	Erigoninae	244
arachnids	Arthropoda	Arachnida	Araneae	Liocranidae	*Agroeca* Westring, 1861	1
arachnids	Arthropoda	Arachnida	Araneae	Linyphiidae	*Agyneta* Hull, 1911	2
arachnids	Arthropoda	Arachnida	Araneae	Lycosidae	*Alopecosa* Simon, 1885	34
arachnids	Arthropoda	Arachnida	Araneae	Araneidae	*Araneus* Clerck, 1758	6
arachnids	Arthropoda	Arachnida	Araneae	Linyphiidae	*Bathyphantes* Menge, 1866	12
arachnids	Arthropoda	Arachnida	Araneae	Linyphiidae	*Centromerita* Dahl, 1912	4
arachnids	Arthropoda	Arachnida	Araneae	Linyphiidae	*Centromerus* Dahl, 1886	10
arachnids	Arthropoda	Arachnida	Araneae	Clubionidae	*Clubiona* Latreille, 1804	103
arachnids	Arthropoda	Arachnida	Araneae	Theridiidae	*Enoplognatha* Pavesi, 1880	2
arachnids	Arthropoda	Arachnida	Araneae	Linyphiidae	*Erigone* Audouin, 1826	15
arachnids	Arthropoda	Arachnida	Araneae	Gnaphosidae	*Haplodrassus* Chamberlin, 1922	5
arachnids	Arthropoda	Arachnida	Araneae	Linyphiidae	*Oedothorax* Bertkau, 1883	27
arachnids	Arthropoda	Arachnida	Araneae	Linyphiidae	*Palliduphantes* Saaristo & Tanasevitch, 2001	33
arachnids	Arthropoda	Arachnida	Araneae	Lycosidae	*Pardosa* Koch, 1847	225
arachnids	Arthropoda	Arachnida	Araneae	Philodromidae	*Philodromus* Walckenaer, 1826	10
arachnids	Arthropoda	Arachnida	Araneae	Linyphiidae	*Pocadicnemis* Simon, 1884	1
arachnids	Arthropoda	Arachnida	Araneae	Linyphiidae	*Porrhomma* Simon, 1884	5
arachnids	Arthropoda	Arachnida	Araneae	Theridiidae	*Robertus* O.P.-Cambridge, 1879	16
arachnids	Arthropoda	Arachnida	Araneae	Salticidae	*Salticus* Latreille, 1804	1
arachnids	Arthropoda	Arachnida	Araneae	Theridiidae	*Theridion* Walckenaer, 1805	6
arachnids	Arthropoda	Arachnida	Araneae	Lycosidae	*Trochosa* Koch, 1847	19
arachnids	Arthropoda	Arachnida	Araneae	Linyphiidae	*Walckenaeria* Blackwall, 1833	7
arachnids	Arthropoda	Arachnida	Araneae	Gnaphosidae	*Zelotes* Gistel, 1848	7
arachnids	Arthropoda	Arachnida	Araneae	Liocranidae	*Agroecabrunnea* (Blackwall, 1833)	5
arachnids	Arthropoda	Arachnida	Araneae	Liocranidae	*Agroecaproxima* (Cambridge, 1871)	2
arachnids	Arthropoda	Arachnida	Araneae	Linyphiidae	*Agynetaconigera* (Cambridge, 1863)	1
arachnids	Arthropoda	Arachnida	Araneae	Linyphiidae	*Agynetadecora* (Cambridge, 1871)	14
arachnids	Arthropoda	Arachnida	Araneae	Linyphiidae	*Agynetarurestris* (Koch, 1836)	2
arachnids	Arthropoda	Arachnida	Araneae	Linyphiidae	*Agynetasaxatilis* (Blackwall, 1844)	41
arachnids	Arthropoda	Arachnida	Araneae	Linyphiidae	*Agynetasubtilis* (Cambridge, 1863)	1
arachnids	Arthropoda	Arachnida	Araneae	Lycosidae	*Alopecosacuneata* (Clerck, 1757)	117
arachnids	Arthropoda	Arachnida	Araneae	Lycosidae	*Alopecosapulverulenta* (Clerck, 1757)	123
arachnids	Arthropoda	Arachnida	Araneae	Theridiidae	*Anelosimusvittatus* (Koch, 1836)	29
arachnids	Arthropoda	Arachnida	Araneae	Anyphaenidae	*Anyphaenaaccentuata* (Walckenaer, 1802)	2
arachnids	Arthropoda	Arachnida	Araneae	Araneidae	*Araneusdiadematus* (Clerck, 1757)	5
arachnids	Arthropoda	Arachnida	Araneae	Araneidae	*Araneusquadratus* (Clerck, 1757)	2
arachnids	Arthropoda	Arachnida	Araneae	Araneidae	*Araneussturmi* (Hahn, 1831)	3
arachnids	Arthropoda	Arachnida	Araneae	Araneidae	*Araneustriguttatus* (Fabricius, 1793)	1
arachnids	Arthropoda	Arachnida	Araneae	Linyphiidae	*Bathyphantesgracilis* (Blackwall, 1841)	314
arachnids	Arthropoda	Arachnida	Araneae	Linyphiidae	*Bathyphantesparvulus* (Westring, 1851)	18
arachnids	Arthropoda	Arachnida	Araneae	Linyphiidae	*Centromeritabicolor* (Blackwall, 1833)	222
arachnids	Arthropoda	Arachnida	Araneae	Linyphiidae	*Centromeritaconcinna* (Thorell, 1875)	13
arachnids	Arthropoda	Arachnida	Araneae	Linyphiidae	*Centromerusdilutus* (Cambridge, 1875)	23
arachnids	Arthropoda	Arachnida	Araneae	Linyphiidae	*Centromeruspabulator* (Cambridge, 1875)	1
arachnids	Arthropoda	Arachnida	Araneae	Linyphiidae	*Centromerusprudens* (Cambridge, 1873)	11
arachnids	Arthropoda	Arachnida	Araneae	Linyphiidae	*Centromerussylvaticus* (Blackwall, 1841)	110
arachnids	Arthropoda	Arachnida	Araneae	Linyphiidae	*Ceratinellabrevipes* (Westring, 1851)	7
arachnids	Arthropoda	Arachnida	Araneae	Linyphiidae	*Ceratinellascabrosa* (Cambridge, 1871)	6
arachnids	Arthropoda	Arachnida	Araneae	Clubionidae	*Clubionabrevipes* (Blackwall, 1841)	5
arachnids	Arthropoda	Arachnida	Araneae	Clubionidae	*Clubionacomta* (Koch, 1839)	9
arachnids	Arthropoda	Arachnida	Araneae	Clubionidae	*Clubionalutescens* (Westring, 1851)	2
arachnids	Arthropoda	Arachnida	Araneae	Clubionidae	*Clubionapallidula* (Clerck, 1757)	31
arachnids	Arthropoda	Arachnida	Araneae	Clubionidae	*Clubionareclusa* (Cambridge, 1863)	37
arachnids	Arthropoda	Arachnida	Araneae	Clubionidae	*Clubionaterrestris* (Westring, 1851)	4
arachnids	Arthropoda	Arachnida	Araneae	Linyphiidae	*Cnephalocotesobscurus* (Blackwall, 1834)	4
arachnids	Arthropoda	Arachnida	Araneae	Theridiidae	*Crustulinaguttata* (Wider, 1834)	2
arachnids	Arthropoda	Arachnida	Araneae	Linyphiidae	*Dicymbiumbrevisetosum* (Locket, 1962)	587
arachnids	Arthropoda	Arachnida	Araneae	Linyphiidae	*Diplocephaluspermixtus* (Cambridge, 1871)	2
arachnids	Arthropoda	Arachnida	Araneae	Linyphiidae	*Diplocephaluspicinus* (Blackwall, 1841)	10
arachnids	Arthropoda	Arachnida	Araneae	Linyphiidae	*Diplostylaconcolor* (Wider, 1834)	11
arachnids	Arthropoda	Arachnida	Araneae	Linyphiidae	*Dismodicusbifrons* (Blackwall, 1841)	2
arachnids	Arthropoda	Arachnida	Araneae	Gnaphosidae	*Drassodespubescens* (Thorell, 1856)	3
arachnids	Arthropoda	Arachnida	Araneae	Gnaphosidae	*Drassylluspusillus* (Koch, 1833)	25
arachnids	Arthropoda	Arachnida	Araneae	Theridiidae	*Enoplognathalatimana* (Hippa & Oksala, 1982)	2
arachnids	Arthropoda	Arachnida	Araneae	Theridiidae	*Enoplognathaovata* (Clerck, 1757)	4
arachnids	Arthropoda	Arachnida	Araneae	Theridiidae	*Enoplognathathoracica* (Hahn, 1833)	56
arachnids	Arthropoda	Arachnida	Araneae	Linyphiidae	*Erigoneatra* (Blackwall, 1833)	211
arachnids	Arthropoda	Arachnida	Araneae	Linyphiidae	*Erigonedentipalpis* (Wider, 1834)	32
arachnids	Arthropoda	Arachnida	Araneae	Linyphiidae	*Erigonellahiemalis* (Blackwall, 1841)	223
arachnids	Arthropoda	Arachnida	Araneae	Linyphiidae	*Erigonellaignobilis* (Cambridge, 1871)	9
arachnids	Arthropoda	Arachnida	Araneae	Salticidae	*Euophrysfrontalis* (Walckenaer, 1802)	9
arachnids	Arthropoda	Arachnida	Araneae	Theridiidae	*Euryopisflavomaculata* (Koch, 1836)	1
arachnids	Arthropoda	Arachnida	Araneae	Linyphiidae	*Floroniabucculenta* (Clerck, 1757)	2
arachnids	Arthropoda	Arachnida	Araneae	Linyphiidae	*Gonatiumrubens* (Blackwall, 1833)	3
arachnids	Arthropoda	Arachnida	Araneae	Linyphiidae	*Gongylidiellumvivum* (Cambridge, 1875)	314
arachnids	Arthropoda	Arachnida	Araneae	Linyphiidae	*Gongylidiumrufipes* (Linnaeus, 1758)	15
arachnids	Arthropoda	Arachnida	Araneae	Gnaphosidae	*Haplodrassussignifer* (Koch, 1839)	26
arachnids	Arthropoda	Arachnida	Araneae	Gnaphosidae	*Haplodrassussilvestris* (Blackwall, 1833)	1
arachnids	Arthropoda	Arachnida	Araneae	Linyphiidae	*Hypommabituberculatum* (Wider, 1834)	1
arachnids	Arthropoda	Arachnida	Araneae	Linyphiidae	*Hypommacornutum* (Blackwall, 1833)	2
arachnids	Arthropoda	Arachnida	Araneae	Hahniidae	*Iberinamontana* (Blackwall, 1841)	10
arachnids	Arthropoda	Arachnida	Opiliones	Phalangiidae	*Laciniusephippiatus* (Koch, 1835)	28
arachnids	Arthropoda	Arachnida	Araneae	Araneidae	*Larinioidescornutus* (Clerck, 1757)	2
arachnids	Arthropoda	Arachnida	Araneae	Araneidae	*Larinioidespatagiatus* (Clerck, 1757)	1
arachnids	Arthropoda	Arachnida	Opiliones	Phalangiidae	*Leiobunumrotundum* (Latreille, 1798)	3
arachnids	Arthropoda	Arachnida	Araneae	Linyphiidae	*Leptorhoptrumrobustum* (Westring, 1851)	55
arachnids	Arthropoda	Arachnida	Araneae	Linyphiidae	*Linyphiatriangularis* (Clerck, 1757)	4
arachnids	Arthropoda	Arachnida	Opiliones	Phalangiidae	*Lophopiliopalpinalis* (Herbst, 1799)	6
arachnids	Arthropoda	Arachnida	Araneae	Linyphiidae	*Macrargusrufus* (Wider, 1834)	19
arachnids	Arthropoda	Arachnida	Araneae	Linyphiidae	*Marominutus* (Cambridge, 1906)	1
arachnids	Arthropoda	Arachnida	Araneae	Linyphiidae	*Masosundevalli* (Westring, 1851)	1
arachnids	Arthropoda	Arachnida	Araneae	Tetragnathidae	*Metellinasegmentata* (Clerck, 1757)	5
arachnids	Arthropoda	Arachnida	Araneae	Gnaphosidae	*Micariapulicaria* (Sundevall, 1831)	3
arachnids	Arthropoda	Arachnida	Araneae	Linyphiidae	*Micrargusherbigradus* (Blackwall, 1854)	1
arachnids	Arthropoda	Arachnida	Araneae	Linyphiidae	*Micrargussubaequalis* (Westring, 1851)	430
arachnids	Arthropoda	Arachnida	Araneae	Linyphiidae	*Micronetaviaria* (Blackwall, 1841)	16
arachnids	Arthropoda	Arachnida	Araneae	Linyphiidae	*Minyrioluspusillus* (Wider, 1834)	1
arachnids	Arthropoda	Arachnida	Araneae	Linyphiidae	*Mioxenablanda* (Simon, 1884)	5
arachnids	Arthropoda	Arachnida	Opiliones	Phalangiidae	*Mitopusmorio* (Fabricius, 1799)	2
arachnids	Arthropoda	Arachnida	Opiliones	Nemastomatidae	*Nemastomalugubre* (Müller, 1776)	16
arachnids	Arthropoda	Arachnida	Pseudoscorpiones	Neobisiidae	*Neobisiumcarcinoides* (Hermann, 1804)	7
arachnids	Arthropoda	Arachnida	Araneae	Linyphiidae	*Nerieneclathrata* (Sundevall, 1830)	4
arachnids	Arthropoda	Arachnida	Araneae	Linyphiidae	*Oedothoraxapicatus* (Blackwall, 1850)	1
arachnids	Arthropoda	Arachnida	Araneae	Linyphiidae	*Oedothoraxfuscus* (Blackwall, 1834)	674
arachnids	Arthropoda	Arachnida	Araneae	Linyphiidae	*Oedothoraxretusus* (Westring, 1851)	1,076
arachnids	Arthropoda	Arachnida	Opiliones	Phalangiidae	*Oligolophushanseni* (Kraepelin, 1896)	9
arachnids	Arthropoda	Arachnida	Opiliones	Phalangiidae	*Oligolophustridens* (Koch, 1836)	261
arachnids	Arthropoda	Arachnida	Opiliones	Phalangiidae	*Opiliosaxatilis* (Koch, 1839)	3
arachnids	Arthropoda	Arachnida	Araneae	Thomisidae	*Ozyptilabrevipes* (Hahn, 1826)	1
arachnids	Arthropoda	Arachnida	Araneae	Thomisidae	*Ozyptilapraticola* (Koch, 1837)	13
arachnids	Arthropoda	Arachnida	Araneae	Thomisidae	*Ozyptilatrux* (Blackwall, 1846)	9
arachnids	Arthropoda	Arachnida	Araneae	Tetragnathidae	*Pachygnathaclercki* (Sundevall, 1823)	320
arachnids	Arthropoda	Arachnida	Araneae	Tetragnathidae	*Pachygnathadegeeri* Sundevall, 1830	1,854
arachnids	Arthropoda	Arachnida	Araneae	Theridiidae	*Paidiscurapallens* (Blackwall, 1834)	8
arachnids	Arthropoda	Arachnida	Araneae	Linyphiidae	*Palliduphantesericaeus* (Blackwall, 1853)	7
arachnids	Arthropoda	Arachnida	Araneae	Linyphiidae	*Palliduphantesinsignis* (Cambridge, 1913)	53
arachnids	Arthropoda	Arachnida	Araneae	Linyphiidae	*Palliduphantespallidus* (Cambridge, 1871)	39
arachnids	Arthropoda	Arachnida	Araneae	Lycosidae	*Pardosaamentata* (Clerck, 1757)	1,234
arachnids	Arthropoda	Arachnida	Araneae	Lycosidae	*Pardosalugubris* (Walckenaer, 1802)	300
arachnids	Arthropoda	Arachnida	Araneae	Lycosidae	*Pardosamonticola* (Clerck, 1757)	3
arachnids	Arthropoda	Arachnida	Araneae	Lycosidae	*Pardosanigriceps* (Thorell, 1856)	1
arachnids	Arthropoda	Arachnida	Araneae	Lycosidae	*Pardosapalustris* (Linnaeus, 1758)	1,548
arachnids	Arthropoda	Arachnida	Araneae	Lycosidae	*Pardosaprativaga* (Koch, 1870)	1,442
arachnids	Arthropoda	Arachnida	Araneae	Lycosidae	*Pardosapullata* (Clerck, 1757)	82
arachnids	Arthropoda	Arachnida	Opiliones	Phalangiidae	*Paroligolophusagrestis* (Meade, 1855)	41
arachnids	Arthropoda	Arachnida	Araneae	Linyphiidae	*Pelecopsisparallela* (Wider, 1834)	3
arachnids	Arthropoda	Arachnida	Araneae	Lycosidae	*Piratahygrophila* (Thorell, 1872)	39
arachnids	Arthropoda	Arachnida	Araneae	Lycosidae	*Piratapiraticus* (Clerck, 1757)	3
arachnids	Arthropoda	Arachnida	Araneae	Linyphiidae	*Pocadicnemisjuncea* (Locket & Millidge, 1953)	10
arachnids	Arthropoda	Arachnida	Araneae	Linyphiidae	*Porrhommacampbelli* (Cambridge, 1894)	22
arachnids	Arthropoda	Arachnida	Araneae	Linyphiidae	*Porrhommamontanum* (Jackson, 1913)	4
arachnids	Arthropoda	Arachnida	Araneae	Linyphiidae	*Porrhommapygmaeum* (Blackwall, 1834)	1
arachnids	Arthropoda	Arachnida	Opiliones	Phalangiidae	*Rilaenatriangularis* (Herbst, 1799)	25
arachnids	Arthropoda	Arachnida	Araneae	Theridiidae	*Robertuslividus* (Blackwall, 1836)	141
arachnids	Arthropoda	Arachnida	Araneae	Linyphiidae	*Saaristoaabnormis* (Blackwall, 1841)	72
arachnids	Arthropoda	Arachnida	Araneae	Salticidae	*Salticuscingulatus* (Panzer, 1797)	1
arachnids	Arthropoda	Arachnida	Araneae	Linyphiidae	*Stemonyphanteslineatus* (Linnaeus, 1758)	1
arachnids	Arthropoda	Arachnida	Araneae	Linyphiidae	*Syedragracilis* (Menge, 1869)	1
arachnids	Arthropoda	Arachnida	Araneae	Linyphiidae	*Tapinocybainsecta* (Koch, 1869)	8
arachnids	Arthropoda	Arachnida	Araneae	Linyphiidae	*Tapinocybapraecox* (Cambridge, 1873)	31
arachnids	Arthropoda	Arachnida	Araneae	Linyphiidae	*Tapinopalongidens* (Wider, 1834)	2
arachnids	Arthropoda	Arachnida	Araneae	Linyphiidae	*Tenuiphantestenuis* (Blackwall, 1852)	14
arachnids	Arthropoda	Arachnida	Araneae	Tetragnathidae	*Tetragnathaextensa* (Linnaeus, 1758)	3
arachnids	Arthropoda	Arachnida	Araneae	Philodromidae	*Thanatusstriatus* (Koch, 1845)	1
arachnids	Arthropoda	Arachnida	Araneae	Theridiidae	*Theridionvarians* (Hahn, 1833)	1
arachnids	Arthropoda	Arachnida	Araneae	Linyphiidae	*Hyreostheniusparasiticus* (Westring, 1851)	1
arachnids	Arthropoda	Arachnida	Araneae	Linyphiidae	*Tisovagans* (Blackwall, 1834)	1,704
arachnids	Arthropoda	Arachnida	Araneae	Lycosidae	*Trochosaruricola* (De Geer, 1778)	25
arachnids	Arthropoda	Arachnida	Araneae	Lycosidae	*Trochosaterricola* Thorell, 1856	229
arachnids	Arthropoda	Arachnida	Araneae	Linyphiidae	*Troxochrusscabriculus* (Westring, 1851)	2
arachnids	Arthropoda	Arachnida	Araneae	Linyphiidae	*Walckenaeriaacuminata* (Blackwall, 1833)	29
arachnids	Arthropoda	Arachnida	Araneae	Linyphiidae	*Walckenaeriaantica* (Wider, 1834)	3
arachnids	Arthropoda	Arachnida	Araneae	Linyphiidae	*Walckenaeriaatrotibialis* (Cambridge, 1878)	3
arachnids	Arthropoda	Arachnida	Araneae	Linyphiidae	*Walckenaeriacucullata* (Koch, 1836)	3
arachnids	Arthropoda	Arachnida	Araneae	Linyphiidae	*Walckenaeriadysderoides* (Wider, 1834)	18
arachnids	Arthropoda	Arachnida	Araneae	Linyphiidae	*Walckenaerianudipalpis* (Westring, 1851)	13
arachnids	Arthropoda	Arachnida	Araneae	Linyphiidae	*Walckenaeriaobtusa* (Blackwall, 1836)	3
arachnids	Arthropoda	Arachnida	Araneae	Linyphiidae	*Walckenaeriavigilax* (Blackwall, 1853)	2
arachnids	Arthropoda	Arachnida	Araneae	Thomisidae	*Xysticuscristatus* (Clerck, 1757)	173
arachnids	Arthropoda	Arachnida	Araneae	Gnaphosidae	*Zeloteselectus* (Koch, 1839)	1
arachnids	Arthropoda	Arachnida	Araneae	Gnaphosidae	*Zeloteslatreillei* (Simon, 1878)	3
arachnids	Arthropoda	Arachnida	Araneae	Gnaphosidae	*Zelotessubterraneus* (Koch, 1833)	8
arachnids	Arthropoda	Arachnida	Araneae	Zoridae	*Zoraspinimana* (Sundevall, 1833)	10
centipedes	Arthropoda	Chilopoda	Geophilomorpha	Geophilidae	*Geophilustruncorum* (Bergsoë & Meinert, 1866)	12
centipedes	Arthropoda	Chilopoda	Lithobiomorpha	Henicopidae	*Lamyctesemarginatus* (Newport, 1844)	64
centipedes	Arthropoda	Chilopoda	Lithobiomorpha	Lithobiidae	*Lithobiusforficatus* (Linnaeus, 1758)	3
centipedes	Arthropoda	Chilopoda	Lithobiomorpha	Lithobiidae	*Lithobiuscrassipes* (Koch, 1862)	10
centipedes	Arthropoda	Chilopoda	Lithobiomorpha	Lithobiidae	*Lithobiusmicrops* (Meinert, 1868)	4
centipedes	Arthropoda	Chilopoda	Geophilomorpha	Geophilidae	*Geophilusflavus* (De Geer, 1778)	2
centipedes	Arthropoda	Chilopoda	Geophilomorpha	Schendylidae	*Schendylanemorensis* (Koch, 1837)	4
millipedes	Arthropoda	Diplopoda	Polydesmida	Polydesmidae	*Brachydesmussuperus* (Latzel, 1884)	7
millipedes	Arthropoda	Diplopoda	Chordeumatida	Craspedosomatidae	*Craspedosomarawlinsi* (Leach, 1814)	18
millipedes	Arthropoda	Diplopoda	Julida	Julidae	*Cylindroiuluscaeruleocinctus* (Wood, 1864)	4
millipedes	Arthropoda	Diplopoda	Julida	Julidae	*Cylindroiuluslatestriatus* (Curtis, 1845)	2
millipedes	Arthropoda	Diplopoda	Julida	Julidae	*Cylindroiuluspunctatus* (Leach, 1815)	4
millipedes	Arthropoda	Diplopoda	Julida	Julidae	*Julusscandinavius* (Latzel, 1884)	5
millipedes	Arthropoda	Diplopoda	Julida	Nemasomatidae	*Nemasomavaricorne* (Koch, 1847)	1
millipedes	Arthropoda	Diplopoda	Julida	Julidae	*Ommatoiulussabulosus* (Linnaeus, 1758)	2
millipedes	Arthropoda	Diplopoda	Polydesmida	Polydesmidae	*Polydesmusdenticulatus* (Koch, 1847)	89
millipedes	Arthropoda	Diplopoda	Julida	Blaniulidae	*Proteroiulusfuscus* (Am Stein, 1857)	6
cicadas	Arthropoda	Insecta	Hemiptera		Auchenorrhyncha	2,089
beetles	Arthropoda	Insecta	Coleoptera	Anobiidae		1
beetles	Arthropoda	Insecta	Coleoptera	Anthicidae		9
beetles	Arthropoda	Insecta	Coleoptera	Byrrhidae		194
beetles	Arthropoda	Insecta	Coleoptera	Cantharidae		298
beetles	Arthropoda	Insecta	Coleoptera	Cerambycidae		3
beetles	Arthropoda	Insecta	Coleoptera	Chrysomelidae		154
beetles	Arthropoda	Insecta	Coleoptera	Chrysomelidae		5
beetles	Arthropoda	Insecta	Coleoptera	Coccinellidae		307
beetles	Arthropoda	Insecta	Coleoptera	Cryptophagidae		60
beetles	Arthropoda	Insecta	Coleoptera	Dermestidae		2
beetles	Arthropoda	Insecta	Coleoptera	Dryopidae		14
beetles	Arthropoda	Insecta	Coleoptera	Histeridae		85
beetles	Arthropoda	Insecta	Coleoptera	Hydrophilidae		1
beetles	Arthropoda	Insecta	Coleoptera	Latridiidae		135
beetles	Arthropoda	Insecta	Coleoptera	Leiodidae		120
beetles	Arthropoda	Insecta	Coleoptera	Leiodidae		30
beetles	Arthropoda	Insecta	Coleoptera	Mordellidae		2
beetles	Arthropoda	Insecta	Coleoptera	Nitidulidae		27
beetles	Arthropoda	Insecta	Coleoptera	Oedemeridae		1
beetles	Arthropoda	Insecta	Coleoptera	Pselaphidae		23
beetles	Arthropoda	Insecta	Coleoptera	Scarabaeidae		56
beetles	Arthropoda	Insecta	Coleoptera	Scirtidae		2
beetles	Arthropoda	Insecta	Coleoptera	Staphylinidae		6,339
beetles	Arthropoda	Insecta	Coleoptera	Throscidae		14
beetles	Arthropoda	Insecta	Coleoptera	Curculionidae	Scolytinae	1
soldier beetle larvae	Arthropoda	Insecta	Coleoptera	Cantharidae	*Cantharis* Linnaeus, 1758	313
soldier beetle larvae	Arthropoda	Insecta	Coleoptera	Cantharidae	*Rhagonycha* Eschscholtz, 1830	62
soldier beetle larvae	Arthropoda	Insecta	Coleoptera	Cantharidae	*Cantharisfusca* (Linnaeus, 1758)	1,824
soldier beetle larvae	Arthropoda	Insecta	Coleoptera	Cantharidae	*Cantharislivida* (Linnaeus, 1758)	74
soldier beetle larvae	Arthropoda	Insecta	Coleoptera	Cantharidae	*Cantharispellucida* (Fabricius, 1792)	23
soldier beetle larvae	Arthropoda	Insecta	Coleoptera	Cantharidae	*Cantharisrustica* (Fallén, 1807)	499
soldier beetle larvae	Arthropoda	Insecta	Coleoptera	Cantharidae	*Rhagonychafulva* (Scopoli, 1763)	3
soldier beetle larvae	Arthropoda	Insecta	Coleoptera	Cantharidae	*Rhagonychalignosa* (Müller, 1764)	48
ground beetles	Arthropoda	Insecta	Coleoptera	Carabidae	*Acupalpusmeridianus* (Linnaeus, 1760)	2
ground beetles	Arthropoda	Insecta	Coleoptera	Carabidae	*Agonumemarginatum* (Gyllenhal, 1827)	12
ground beetles	Arthropoda	Insecta	Coleoptera	Carabidae	*Agonummuelleri* (Herbst, 1784)	31
ground beetles	Arthropoda	Insecta	Coleoptera	Carabidae	*Agonumsexpunctatum* (Linnaeus, 1758)	2
ground beetles	Arthropoda	Insecta	Coleoptera	Carabidae	*Amaraaenea* (De Geer, 1774)	91
ground beetles	Arthropoda	Insecta	Coleoptera	Carabidae	*Amaraapricaria* (Paykull, 1790)	1
ground beetles	Arthropoda	Insecta	Coleoptera	Carabidae	*Amaracommunis* (Panzer, 1797)	643
ground beetles	Arthropoda	Insecta	Coleoptera	Carabidae	*Amaraequestris* (Duftschmid, 1812)	1
ground beetles	Arthropoda	Insecta	Coleoptera	Carabidae	*Amarafamiliaris* (Duftschmid, 1812)	72
ground beetles	Arthropoda	Insecta	Coleoptera	Carabidae	*Amaralunicollis* (Schiødte, 1837)	219
ground beetles	Arthropoda	Insecta	Coleoptera	Carabidae	*Amaranitida* (Sturm, 1825)	1
ground beetles	Arthropoda	Insecta	Coleoptera	Carabidae	*Amaraplebeja* (Gyllenhal, 1810)	132
ground beetles	Arthropoda	Insecta	Coleoptera	Carabidae	*Amarasimilata* (Gyllenhal, 1810)	8
ground beetles	Arthropoda	Insecta	Coleoptera	Carabidae	*Amaraspreta* (Dejean, 1831)	1
ground beetles	Arthropoda	Insecta	Coleoptera	Carabidae	*Anchomenusdorsalis* (Pontoppidan, 1763)	7
ground beetles	Arthropoda	Insecta	Coleoptera	Carabidae	*Anisodactylusbinotatus* (Fabricius, 1787)	208
ground beetles	Arthropoda	Insecta	Coleoptera	Carabidae	*Badisterbullatus* (Schrank, 1798)	2
ground beetles	Arthropoda	Insecta	Coleoptera	Carabidae	*Badisterunipustulatus* (Bonelli, 1813)	1
ground beetles	Arthropoda	Insecta	Coleoptera	Carabidae	*Bembidionlampros* (Herbst, 1784)	70
ground beetles	Arthropoda	Insecta	Coleoptera	Carabidae	*Bembidionproperans* (Stephens, 1828)	4
ground beetles	Arthropoda	Insecta	Coleoptera	Carabidae	*Bembidiontetracolum* (Say, 1823)	4
ground beetles	Arthropoda	Insecta	Coleoptera	Carabidae	*Bradycellusharpalinus* (Audinet-Serville, 1821)	216
ground beetles	Arthropoda	Insecta	Coleoptera	Carabidae	*Bradycellusruficollis* (Stephens, 1828)	1
ground beetles	Arthropoda	Insecta	Coleoptera	Carabidae	*Calathuscinctus* (Motschulsky, 1850)	1
ground beetles	Arthropoda	Insecta	Coleoptera	Carabidae	*Calathusfuscipes* (Goeze, 1777)	9
ground beetles	Arthropoda	Insecta	Coleoptera	Carabidae	*Calathusmelanocephalus* (Linnaeus, 1758)	171
ground beetles	Arthropoda	Insecta	Coleoptera	Carabidae	*Calathusrotundicollis* (Dejean, 1828)	1
ground beetles	Arthropoda	Insecta	Coleoptera	Carabidae	*Carabusgranulatus* (Linnaeus, 1758)	13
ground beetles	Arthropoda	Insecta	Coleoptera	Carabidae	*Carabusnemoralis* (Müller, 1764)	49
ground beetles	Arthropoda	Insecta	Coleoptera	Carabidae	*Chlaeniusnigricornis* (Fabricius, 1787)	6
ground beetles	Arthropoda	Insecta	Coleoptera	Carabidae	*Clivinafossor* (Fabricius, 1787)	133
ground beetles	Arthropoda	Insecta	Coleoptera	Carabidae	*Cychruscaraboides* (Linnaeus, 1758)	18
ground beetles	Arthropoda	Insecta	Coleoptera	Carabidae	*Dicheirotrichusplacidus* (Gyllenhal, 1827)	7
ground beetles	Arthropoda	Insecta	Coleoptera	Carabidae	*Harpaluslaevipes* (Zetterstedt, 1828)	16
ground beetles	Arthropoda	Insecta	Coleoptera	Carabidae	*Harpaluslatus* (Linnaeus, 1758)	455
ground beetles	Arthropoda	Insecta	Coleoptera	Carabidae	*Harpalusrufipes* (De Geer, 1774)	178
ground beetles	Arthropoda	Insecta	Coleoptera	Carabidae	*Harpalussolitaris* (De Geer, 1774)	1
ground beetles	Arthropoda	Insecta	Coleoptera	Carabidae	*Leistusterminatus* (Panzer, 1793)	25
ground beetles	Arthropoda	Insecta	Coleoptera	Carabidae	*Loricerapilicornis* (Fabricius, 1775)	9
ground beetles	Arthropoda	Insecta	Coleoptera	Carabidae	*Nebriabrevicollis* (Fabricius, 1792)	47
ground beetles	Arthropoda	Insecta	Coleoptera	Carabidae	*Nebriasalina* (Fairmaire & Laboulbène, 1854)	2
ground beetles	Arthropoda	Insecta	Coleoptera	Carabidae	*Notiophilusbiguttatus* (Fabricius, 1779)	2
ground beetles	Arthropoda	Insecta	Coleoptera	Carabidae	*Notiophiluspalustris* (Duftschmid, 1812)	19
ground beetles	Arthropoda	Insecta	Coleoptera	Carabidae	*Notiophilusrufipes* (Curtis, 1829)	1
ground beetles	Arthropoda	Insecta	Coleoptera	Carabidae	*Ophonusrufibarbis* (Fabricius, 1792)	181
ground beetles	Arthropoda	Insecta	Coleoptera	Carabidae	*Oxypselaphusobscurus* (Herbst, 1784)	26
ground beetles	Arthropoda	Insecta	Coleoptera	Carabidae	*Panagaeuscruxmajor* (Linnaeus, 1758)	3
ground beetles	Arthropoda	Insecta	Coleoptera	Carabidae	*Patrobusatrorufus* (Ström, 1768)	3
ground beetles	Arthropoda	Insecta	Coleoptera	Carabidae	*Philorhizusmelanocephalus* (Dejean, 1825)	3
ground beetles	Arthropoda	Insecta	Coleoptera	Carabidae	*Platynusassimilis* (Paykull, 1790)	4
ground beetles	Arthropoda	Insecta	Coleoptera	Carabidae	*Poecilusversicolor* (Sturm, 1824)	1,975
ground beetles	Arthropoda	Insecta	Coleoptera	Carabidae	*Pterostichusdiligens* (Sturm, 1824)	2
ground beetles	Arthropoda	Insecta	Coleoptera	Carabidae	*Pterostichusmelanarius* (Illiger, 1798)	285
ground beetles	Arthropoda	Insecta	Coleoptera	Carabidae	*Pterostichusniger* (Schaller, 1783)	31
ground beetles	Arthropoda	Insecta	Coleoptera	Carabidae	*Pterostichusnigrita* (Paykull, 1790)	23
ground beetles	Arthropoda	Insecta	Coleoptera	Carabidae	*Pterostichusoblongopunctatus* (Fabricius, 1787)	35
ground beetles	Arthropoda	Insecta	Coleoptera	Carabidae	*Pterostichusstrenuus* (Panzer, 1796)	356
ground beetles	Arthropoda	Insecta	Coleoptera	Carabidae	*Pterostichusvernalis* (Panzer, 1796)	156
ground beetles	Arthropoda	Insecta	Coleoptera	Carabidae	*Stenolophusteutonus* (Schrank, 1781)	1
ground beetles	Arthropoda	Insecta	Coleoptera	Carabidae	*Syntomusfoveatus* (Geoffroy, 1785)	1
ground beetles	Arthropoda	Insecta	Coleoptera	Carabidae	*Syntomustruncatellus* (Linnaeus, 1760)	10
ground beetles	Arthropoda	Insecta	Coleoptera	Carabidae	*Synuchusvivalis* (Illiger, 1798)	66
ground beetles	Arthropoda	Insecta	Coleoptera	Carabidae	*Trechoblemusmicros* (Herbst, 1784)	8
ground beetles	Arthropoda	Insecta	Coleoptera	Carabidae	*Trechusobtusus* (Erichson, 1837)	334
ground beetle larvae	Arthropoda	Insecta	Coleoptera	Carabidae	*Agonum* Bonelli, 1810	1
ground beetle larvae	Arthropoda	Insecta	Coleoptera	Carabidae	*Amara* Bonelli, 1810	18
ground beetle larvae	Arthropoda	Insecta	Coleoptera	Carabidae	*Cychrus* Fabricius, 1794	3
ground beetle larvae	Arthropoda	Insecta	Coleoptera	Carabidae	*Harpalus* Latreille, 1802	6
ground beetle larvae	Arthropoda	Insecta	Coleoptera	Carabidae	*Leistus* Frölich, 1799	6
ground beetle larvae	Arthropoda	Insecta	Coleoptera	Carabidae	*Agonummuelleri* (Herbst, 1784)	2
ground beetle larvae	Arthropoda	Insecta	Coleoptera	Carabidae	*Agonumsexpunctatum* (Linnaeus, 1758)	7
ground beetle larvae	Arthropoda	Insecta	Coleoptera	Carabidae	*Amaraaenea* (De Geer, 1774)	1
ground beetle larvae	Arthropoda	Insecta	Coleoptera	Carabidae	*Amaraaulica* (Panzer, 1796)	1
ground beetle larvae	Arthropoda	Insecta	Coleoptera	Carabidae	*Amaraconvexior* (Stephens, 1828)	19
ground beetle larvae	Arthropoda	Insecta	Coleoptera	Carabidae	*Amarafamiliaris* (Duftschmid, 1812)	2
ground beetle larvae	Arthropoda	Insecta	Coleoptera	Carabidae	*Anisodactylusbinotatus* (Fabricius, 1787)	6
ground beetle larvae	Arthropoda	Insecta	Coleoptera	Carabidae	*Carabusgranulatus* (Linnaeus, 1758)	9
ground beetle larvae	Arthropoda	Insecta	Coleoptera	Carabidae	*Carabusnemoralis* (Müller, 1764)	83
ground beetle larvae	Arthropoda	Insecta	Coleoptera	Carabidae	*Clivinafossor* (Fabricius, 1787)	19
ground beetle larvae	Arthropoda	Insecta	Coleoptera	Carabidae	*Cychruscaraboides* (Linnaeus, 1758)	1
ground beetle larvae	Arthropoda	Insecta	Coleoptera	Carabidae	*Harpaluslatus* (Linnaeus, 1758)	11
ground beetle larvae	Arthropoda	Insecta	Coleoptera	Carabidae	*Harpalusrufipes* (De Geer, 1774)	35
ground beetle larvae	Arthropoda	Insecta	Coleoptera	Carabidae	*Loricerapilicornis* (Fabricius, 1775)	5
ground beetle larvae	Arthropoda	Insecta	Coleoptera	Carabidae	*Nebriabrevicollis* (Fabricius, 1792)	38
ground beetle larvae	Arthropoda	Insecta	Coleoptera	Carabidae	*Notiophiluspalustris* (Duftschmid, 1812)	14
ground beetle larvae	Arthropoda	Insecta	Coleoptera	Carabidae	*Ophonusrupicola* (Sturm, 1818)	29
ground beetle larvae	Arthropoda	Insecta	Coleoptera	Carabidae	*Patrobusatrorufus* (Ström, 1768)	6
ground beetle larvae	Arthropoda	Insecta	Coleoptera	Carabidae	*Poeciluscupreus* (Linnaeus, 1758)	1
ground beetle larvae	Arthropoda	Insecta	Coleoptera	Carabidae	*Poecilusversicolor* (Sturm, 1824)	6
ground beetle larvae	Arthropoda	Insecta	Coleoptera	Carabidae	*Pterostichusmelanarius* (Illiger, 1798)	12
weevils	Arthropoda	Insecta	Coleoptera	Curculionidae	*Rhamphus* Clairville, 1798	8
weevils	Arthropoda	Insecta	Coleoptera	Brentidae	*Apioncruentatum* (Walton, 1844)	53
weevils	Arthropoda	Insecta	Coleoptera	Brentidae	*Apionfrumentarium* (Linnaeus, 1758)	2
weevils	Arthropoda	Insecta	Coleoptera	Curculionidae	*Archariuspyrrhoceras* (Marsham, 1802)	6
weevils	Arthropoda	Insecta	Coleoptera	Curculionidae	*Barynotusobscurus* (Fabricius, 1775)	8
weevils	Arthropoda	Insecta	Coleoptera	Brentidae	*Betulapionsimile* (Kirby, 1811)	19
weevils	Arthropoda	Insecta	Coleoptera	Curculionidae	*Brachyperazoilus* (Scopoli, 1763)	2
weevils	Arthropoda	Insecta	Coleoptera	Curculionidae	*Ceutorhynchuscontractus* (Marsham, 1802)	1
weevils	Arthropoda	Insecta	Coleoptera	Curculionidae	*Ceutorhynchustyphae* (Herbst, 1795)	1
weevils	Arthropoda	Insecta	Coleoptera	Curculionidae	*Chlorophanusviridis* (Linnaeus, 1758)	24
weevils	Arthropoda	Insecta	Coleoptera	Curculionidae	*Coeliodestransversealbofasciatus* (Goeze, 1777)	8
weevils	Arthropoda	Insecta	Coleoptera	Curculionidae	*Curculioglandium* (Marsham, 1802)	2
weevils	Arthropoda	Insecta	Coleoptera	Curculionidae	*Curculiovenosus* (Gravenhorst, 1807)	2
weevils	Arthropoda	Insecta	Coleoptera	Attelabidae	*Deporausbetulae* (Linnaeus, 1758)	1
weevils	Arthropoda	Insecta	Coleoptera	Curculionidae	*Dorytomusmelanophthalmus* (Paykull, 1792)	1
weevils	Arthropoda	Insecta	Coleoptera	Curculionidae	*Dorytomustaeniatus* (Fabricius, 1781)	1
weevils	Arthropoda	Insecta	Coleoptera	Curculionidae	*Dorytomustortrix* (Linnaeus, 1760)	3
weevils	Arthropoda	Insecta	Coleoptera	Curculionidae	*Glocianuspunctiger* (Sahlberg, 1835)	16
weevils	Arthropoda	Insecta	Coleoptera	Curculionidae	*Hyperaplantaginis* (De Geer, 1775)	1
weevils	Arthropoda	Insecta	Coleoptera	Brentidae	*Ischnopterapionloti* (Kirby, 1808)	1
weevils	Arthropoda	Insecta	Coleoptera	Brentidae	*Ischnopterapionvirens* (Herbst, 1797)	59
weevils	Arthropoda	Insecta	Coleoptera	Curculionidae	*Magdalisflavicornis* (Gyllenhal, 1835)	1
weevils	Arthropoda	Insecta	Coleoptera	Curculionidae	*Mecinuspyraster* (Herbst, 1795)	3
weevils	Arthropoda	Insecta	Coleoptera	Curculionidae	*Orchestesbetuleti* (Panzer, 1795)	1
weevils	Arthropoda	Insecta	Coleoptera	Curculionidae	*Orchestesquercus* (Linnaeus, 1758)	2
weevils	Arthropoda	Insecta	Coleoptera	Curculionidae	*Otiorhynchusraucus* (Fabricius, 1777)	41
weevils	Arthropoda	Insecta	Coleoptera	Curculionidae	*Otiorhynchussingularis* (Linnaeus, 1767)	3
weevils	Arthropoda	Insecta	Coleoptera	Brentidae	*Perapioncurtirostre* (Germar, 1817)	15
weevils	Arthropoda	Insecta	Coleoptera	Brentidae	*Perapionviolaceum* (Kirby, 1808)	14
weevils	Arthropoda	Insecta	Coleoptera	Curculionidae	*Phyllobiusargentatus* (Linnaeus, 1758)	6
weevils	Arthropoda	Insecta	Coleoptera	Curculionidae	*Phyllobiusmaculicornis* (Germar, 1824)	4
weevils	Arthropoda	Insecta	Coleoptera	Curculionidae	*Phyllobiuspomaceus* Gyllenhal, 1834	1
weevils	Arthropoda	Insecta	Coleoptera	Curculionidae	*Phyllobiuspyri* (Linnaeus, 1758)	7
weevils	Arthropoda	Insecta	Coleoptera	Curculionidae	*Phyllobiusvirideaeris* (Laicharting, 1781)	13
weevils	Arthropoda	Insecta	Coleoptera	Curculionidae	*Polydrususcervinus* (Linnaeus, 1758)	21
weevils	Arthropoda	Insecta	Coleoptera	Curculionidae	*Polydrususformosus* (Mayer, 1779)	2
weevils	Arthropoda	Insecta	Coleoptera	Brentidae	*Protapionfulvipes* (Geoffroy, 1785)	8
weevils	Arthropoda	Insecta	Coleoptera	Brentidae	*Protapionnigritarse* (Kirby, 1808)	1
weevils	Arthropoda	Insecta	Coleoptera	Curculionidae	*Rhamphusoxya* (Marsham, 1802)	1
weevils	Arthropoda	Insecta	Coleoptera	Curculionidae	*Rhinoncuscastor* (Fabricius, 1793)	1
weevils	Arthropoda	Insecta	Coleoptera	Curculionidae	*Rhinoncuspericarpius* (Linnaeus, 1758)	62
weevils	Arthropoda	Insecta	Coleoptera	Curculionidae	*Sitonahispidulus* (Fabricius, 1777)	4
weevils	Arthropoda	Insecta	Coleoptera	Curculionidae	*Sitonalepidus* (Gyllenhal, 1834)	51
weevils	Arthropoda	Insecta	Coleoptera	Curculionidae	*Strophosomamelanogrammum* (Forster, 1771)	9
weevils	Arthropoda	Insecta	Coleoptera	Curculionidae	*Tachyergesstigma* (Germar, 1821)	1
weevils	Arthropoda	Insecta	Coleoptera	Curculionidae	*Trachodeshispidus* (Linnaeus, 1758)	1
weevils	Arthropoda	Insecta	Coleoptera	Curculionidae	*Trichosirocalustroglodytes* (Fabricius, 1787)	1
weevils	Arthropoda	Insecta	Coleoptera	Curculionidae	*Tychiuspicirostris* (Fabricius, 1787)	2
click beetles	Arthropoda	Insecta	Coleoptera	Elateridae		18
click beetles	Arthropoda	Insecta	Coleoptera	Elateridae	*Agrioteslineatus* (Linnaeus, 1767)	12
click beetles	Arthropoda	Insecta	Coleoptera	Elateridae	*Agriotesobscurus* (Linnaeus, 1758)	412
click beetles	Arthropoda	Insecta	Coleoptera	Elateridae	*Athoushaemorrhoidalis* (Fabricius, 1801)	33
click beetles	Arthropoda	Insecta	Coleoptera	Elateridae	*Dalopiusmarginatus* (Linnaeus, 1758)	7
click beetles	Arthropoda	Insecta	Coleoptera	Elateridae	*Hemicrepidiusniger* (Linnaeus, 1758)	26
wireworms	Arthropoda	Insecta	Coleoptera	Elateridae	*Agriotes* Eschscholtz, 1829	255
wireworms	Arthropoda	Insecta	Coleoptera	Elateridae	*Actenicerussjaelandicus* (Müller, 1764)	1
wireworms	Arthropoda	Insecta	Coleoptera	Elateridae	*Dalopiusmarginatus* (Linnaeus, 1758)	2
wireworms	Arthropoda	Insecta	Coleoptera	Elateridae	*Denticollislinearis* (Linnaeus, 1758)	49
wireworms	Arthropoda	Insecta	Coleoptera	Elateridae	*Hemicrepidiusniger* (Linnaeus, 1758)	10
silphid larvae	Arthropoda	Insecta	Coleoptera	Silphidae	*Phosphugaatrata* (Linnaeus, 1758)	20
silphid larvae	Arthropoda	Insecta	Coleoptera	Silphidae	*Silphaobscura* (Linnaeus, 1758)	642
rove beetle larvae	Arthropoda	Insecta	Coleoptera	Staphylinidae	Staphylinidae	2
rove beetle larvae	Arthropoda	Insecta	Coleoptera	Staphylinidae	Aleocharinae	5
rove beetle larvae	Arthropoda	Insecta	Coleoptera	Staphylinidae	Staphylininae	3
rove beetle larvae	Arthropoda	Insecta	Coleoptera	Staphylinidae	Staphylininae	112
rove beetle larvae	Arthropoda	Insecta	Coleoptera	Staphylinidae	Steninae	10
rove beetle larvae	Arthropoda	Insecta	Coleoptera	Staphylinidae	*Creophilus* Samouelle, 1819	2
rove beetle larvae	Arthropoda	Insecta	Coleoptera	Staphylinidae	*Gyrohypnus* Samouelle, 1819	57
rove beetle larvae	Arthropoda	Insecta	Coleoptera	Staphylinidae	*Lathrobium* Gravenhorst, 1802	3
rove beetle larvae	Arthropoda	Insecta	Coleoptera	Staphylinidae	*Leptacinus* Erichson, 1839	24
rove beetle larvae	Arthropoda	Insecta	Coleoptera	Staphylinidae	*Paederus* Fabricius, 1775	14
rove beetle larvae	Arthropoda	Insecta	Coleoptera	Staphylinidae	*Quedius* Stephens, 1829	108
rove beetle larvae	Arthropoda	Insecta	Coleoptera	Staphylinidae	*Tachinus* Gravenhorst, 1802	504
rove beetle larvae	Arthropoda	Insecta	Coleoptera	Staphylinidae	*Xantholinus* Dejean, 1821	58
earwigs	Arthropoda	Insecta	Dermaptera			962
dipterans	Arthropoda	Insecta	Diptera		Nematocera	11,456
dipterans	Arthropoda	Insecta	Diptera	Agromyzidae		19
dipterans	Arthropoda	Insecta	Diptera	Anthomyzidae		3
dipterans	Arthropoda	Insecta	Diptera	Asilidae		13
dipterans	Arthropoda	Insecta	Diptera	Asteiidae		37
dipterans	Arthropoda	Insecta	Diptera	Bibionidae		52
dipterans	Arthropoda	Insecta	Diptera	Calliphoridae		278
dipterans	Arthropoda	Insecta	Diptera	Carnidae		2,199
dipterans	Arthropoda	Insecta	Diptera	Chloropidae		1,121
dipterans	Arthropoda	Insecta	Diptera	Dolichopodidae		455
dipterans	Arthropoda	Insecta	Diptera	Drosophilidae		1,161
dipterans	Arthropoda	Insecta	Diptera	Empididae		525
dipterans	Arthropoda	Insecta	Diptera	Ephydridae		2
dipterans	Arthropoda	Insecta	Diptera	Heleomyzidae		208
dipterans	Arthropoda	Insecta	Diptera	Lauxaniidae		13
dipterans	Arthropoda	Insecta	Diptera	Lonchopteridae		83
dipterans	Arthropoda	Insecta	Diptera	Megamerinidae		7
dipterans	Arthropoda	Insecta	Diptera	Micropezidae		1
dipterans	Arthropoda	Insecta	Diptera	Milichiidae		2
dipterans	Arthropoda	Insecta	Diptera	Muscidae		1,766
dipterans	Arthropoda	Insecta	Diptera	Opomyzidae		99
dipterans	Arthropoda	Insecta	Diptera	Phoridae		1,630
dipterans	Arthropoda	Insecta	Diptera	Pipunculidae		6
dipterans	Arthropoda	Insecta	Diptera	Psilidae		10
dipterans	Arthropoda	Insecta	Diptera	Rhagionidae		19
dipterans	Arthropoda	Insecta	Diptera	Sarcophagidae		378
dipterans	Arthropoda	Insecta	Diptera	Scathophagidae		538
dipterans	Arthropoda	Insecta	Diptera	Sepsidae		381
dipterans	Arthropoda	Insecta	Diptera	Sphaeroceridae		862
dipterans	Arthropoda	Insecta	Diptera	Syrphidae		112
dipterans	Arthropoda	Insecta	Diptera	Tabanidae		7
dipterans	Arthropoda	Insecta	Diptera	Tachinidae		74
dipterans	Arthropoda	Insecta	Diptera	Therevidae		13
dipterans	Arthropoda	Insecta	Diptera	Tipulidae		93
crane fly larvae	Arthropoda	Insecta	Diptera	Tipulidae		367
dipteran larvae	Arthropoda	Insecta	Diptera		Brachycera	329
dipteran larvae	Arthropoda	Insecta	Diptera		Nematocera	19
true bugs	Arthropoda	Insecta	Hemiptera		Heteropthera	446
narrow-waisted hymenopterans	Arthropoda	Insecta	Hymenoptera		Apocrita	6,126
sawfly larvae	Arthropoda	Insecta	Hymenoptera		Symphyta	16
moths	Arthropoda	Insecta	Lepidoptera		Heterocera	209
caterpillars	Arthropoda	Insecta	Lepidoptera			301
grasshoppers	Arthropoda	Insecta	Orthoptera		Caelifera	13
isopods	Arthropoda	Malacostraca	Isopoda	Ligiidae	*Ligidiumhypnorum* (Cuvier, 1792)	6
isopods	Arthropoda	Malacostraca	Isopoda	Oniscidae	*Oniscusasellus* (Linnaeus, 1758)	2
isopods	Arthropoda	Malacostraca	Isopoda	Philosciidae	*Philosciamuscorum* (Scopoli, 1763)	240
isopods	Arthropoda	Malacostraca	Isopoda	Porcellionidae	*Porcellioscaber* (Latreille, 1804)	1
isopods	Arthropoda	Malacostraca	Isopoda	Trichoniscidae	*Trichoniscuspusillus* (Brandt, 1833)	37
