# Supplementary material for: Sampling data of macro-invertebrates collected in grasslands under restoration succession in a lowland stream-valley system
Source: Biodivers Data J. 2024 Jul 23;12:e125462. doi: 10.3897/BDJ.12.e125462 (PMC11292119; doi:10.3897/BDJ.12.e125462)
Supplement: Supplementary material 1 — Supplementary material Drentsche Aa 1996 [file bdj-12-e125462-s001.docx]

Supplementary material Drentse Aa: pictures and vegetation

The research was carried out in the Drentse Aa region, which is situated in the North-eastern part of the Netherlands. The sampling has been performed in 1996 from 11 March until 23 December. Fields O, B, C and K were agricultural fields in which fertilization stopped in the years, 1988, 1985, 1972 and 1967 respectively.

| Table S.1 plant species in the fields O, B, C and K during 1996. The numbers are % cover | | | | |
| --- | --- | --- | --- | --- |
| **Plot** | **O** | **B** | **C** | **K** |
| *Holcus lanatus* | 12 | 76 | 4 | 0 |
| *Lolium perenne* | 36 | 0 | 0 | 0 |
| *Agrostis stolonifera* | 36 | 7 | 0 | 0 |
| *Poa sp.* | 2 | 0 | 0 | 0 |
| *Ranunculus repens* | 13 | 9 | 0 | 0 |
| *Dactylis glomerata* | 0 | 2 | 0 | 0 |
| *Cerastium fontanum* | 1 | 0 | 0 | 0 |
| *Taraxacum officinale* | 0 | 1 | 0 | 0 |
| *Festuca rubra* | 0 | 0 | 32 | 38 |
| *Agrostis capillaris* | 0 | 0 | 62 | 18 |
| *Anthoxanthum odoratum* | 0 | 0 | 0 | 3 |
| *Galium saxatile* | 0 | 0 | 0 | 17 |
| *Rumex acetosa* | 0 | 1 | 1 | 0 |
| *Plantago lanceolata* | 0 | 0 | 1 | 0 |
| *Anthriscus sylvestris* | 0 | 1 | 0 | 0 |
| Other | 0 | 2 | 0 | 0 |
| Total | 100 | 100 | 100 | 76 |

We have visited the area on 2 July 2021 and have registered the GPS coordinates of the fields and the vegetation composition as presence only data.

| Table S.2 plant species in field O (latitude 53.0449 north; longitude 6.6799 east) on 2 July 2021 | | |
| --- | --- | --- |
| **English name** | **Dutch name** | **Latin name** |
| Crested Dog's-tail | Kamgras | *Cynosurus cristatus* |
| Greater Yellow-rattle | Grote Ratelaar | *Rhinanthus angustifolius* |
| Common Starwort | Grasmuur | *Stellaria graminea* |
| Ribwort Plantain | Smalle Weegbree | *Plantago lanceolata* |
| Cat Grass | Kropaar | *Dactylis glomerata* |
| Yorkshire Fog | Gestreepte Witbol | *Holcus lanatus* |
| Sweet Vernal Grass | Reukgras | *Anthoxanthum odoratum* |
| Rough Bluegrass | Ruw Beemdgras | *Poa trivialis* |
| Cow Parsley | Fluitenkruid | *Anthriscus sylvestris* |
| Meadow buttercup | Scherpe Boterbloem | *Ranunculus acris* |
|  | Knopktuid | *Galinsoga* |
| Sorrel | Veldzuring | *Rumex acetosa* |
|  | Brunel | *Prunella vulgaris* |
|  | Leeuwentand | *Leontodon spec* |
|  | Kruisbladwalstro (?) | *Galium cruciata* |
|  | Knopkruid (?) | *Galinsoga* |

| Table S.3 plant species in field B (latitude 53.0476 north; longitude 6.6759 east) on 2 July 2021 | | |
| --- | --- | --- |
| **English name** | **Dutch name** | **Latin name** |
| Bladen met puntjes; witte bloem | Glad walstro (?) of echt walstro | *Galium mollugo/ Galium Verum* |
| Common groundsel | Klein Kruiskruid | *Senecio vulgaris* |
| Cow Parsley | Fluitenkruid | *Anthriscus sylvestris* |
| Catsear | Gewoon Biggenkruid | *Hypochaeris radicata* |
| Ribwort Plantain | Smalle Weegbree | *Plantago lanceolata* |
|  | Schapengras |  |
| Rough Bluegrass | Ruw Beemdgras | *Poa trivialis* |
| Yorkshire Fog | Gestreepte Witbol | *Holcus lanatus* |
| Cat Grass | Kropaar | *Dactylis glomerata* |
|  | Kleefkruid | *Galium aparine* |
| Sorrel | Veldzuring | *Rumex acetosa* |
| Marsh Thistle | Kale Jonker | *Cirsium palustre* |
| Greater Yellow-rattle | Grote Ratelaar | *Rhinanthus angustifolius* |
| Common Starwort | Grasmuur | *Stellaria graminea* |

| Table S.4 plant species in field C (latitude 53.0502 north; longitude 6.6687 east) on 2 July 2021 | | |
| --- | --- | --- |
| **English name** | **Dutch name** | **Latin name** |
| Common Rush | Pitrus | *Juncus effusus* |
| Sweet Vernal Grass | Reukgras | *Anthoxanthum odoratum* |
|  | Glad walstro (?) of moeraswalstro | *Galium mollugo/ Galium palustre* |
| Rough Bluegrass | Ruw Beemdgras | *Poa trivialis* |
| Sorrel | Veldzuring | *Rumex acetosa* |
| Common Woodrush | Veelbloemige veldbies | *Luzula multiflora* |
|  | Blauwe zegge | *Carex panicea* |
|  | Zwarte zegge | *Carex nigra* |
| Oval sedge | Hazenzegge | *Carex leporina* |
| Marsh Thistle | Kale Jonker | *Cirsium palustre* |
| Springy Turf-moss | Haakmos | *Rhytidiadelphus squarrosus* |
| Bird's foot trefoil | Rolklaver | *Lotus corniculatus* |
| Meadowsweet | Moerasspirea | *Filipendula ulmaria* |
| Catsear | Gewoon Biggenkruid | *Hypochaeris radicata* |

| Table S.5 plant species in field K (latitude 53.0479 north; longitude 6.6661 east) on 2 July 2021 | | |
| --- | --- | --- |
| **English name** | **Dutch name** | **Latin name** |
| Springy Turf-moss | Haakmos | *Rhytidiadelphus squarrosus* |
| Red Fescue | Rood Zwenkgras | *Festuca rubra* |
| Common Tormentil | Tormentil | *Potentilla erecta* |
| Greater Yellow-rattle | Grote Ratelaar | *Rhinanthus angustifolius* |
| Common Rush | Pitrus | *Juncus effusus* |
| Heath Rush | Trekrus | *Juncus squarrosus* |
| Feld Woodrush | Gewone Veldbies | *Luzula campestris* |
|  | Glad walstro (?) of moeraswalstro | *Galium mollugo/ Galium palustre* |
| Sweet Vernal Grass | Reukgras | *Anthoxanthum odoratum* |
| Oval sedge | Hazenzegge | *Carex leporina* |

Field O pictures on 2 July 2021

| 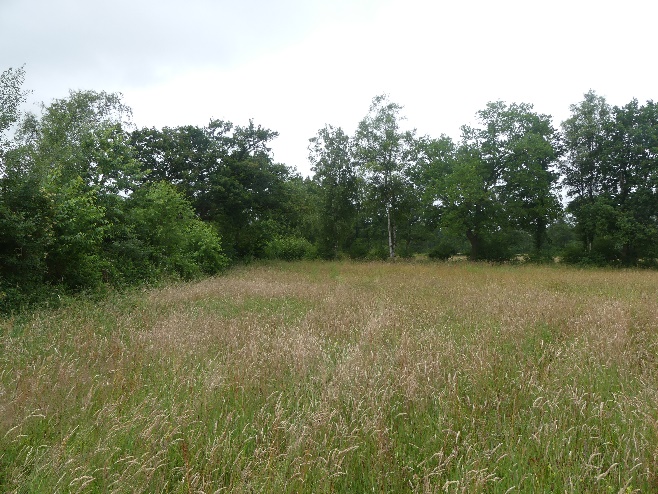 | 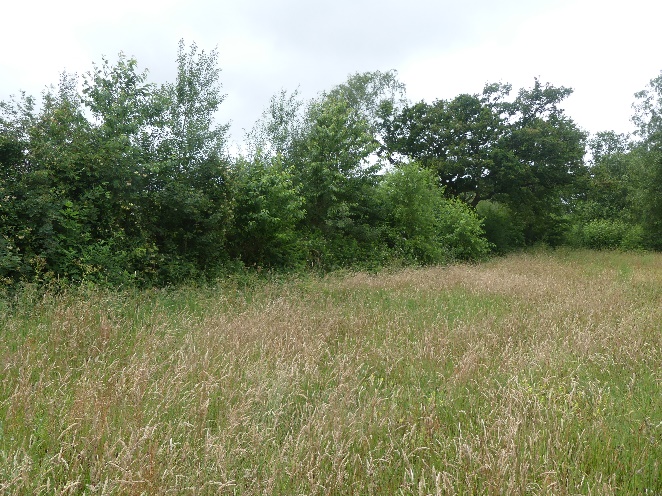 |
| --- | --- |
| 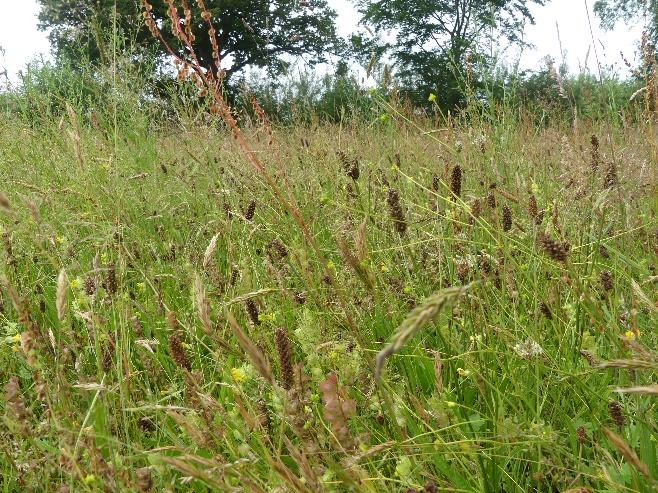 | 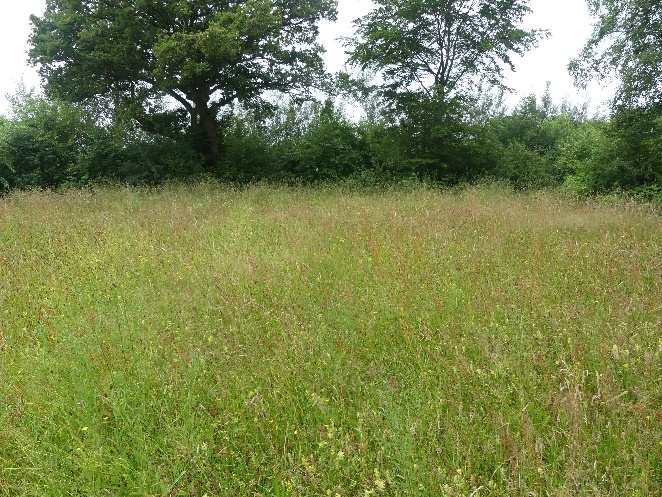 |

Field B pictures on 2 July 2021

| 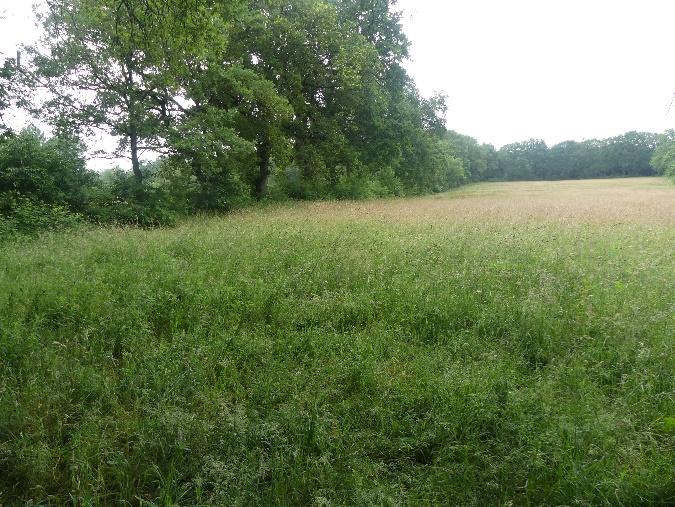 | 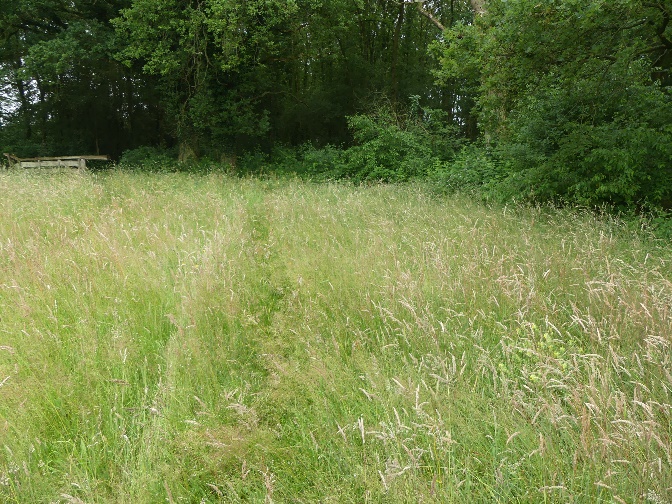 |
| --- | --- |
| 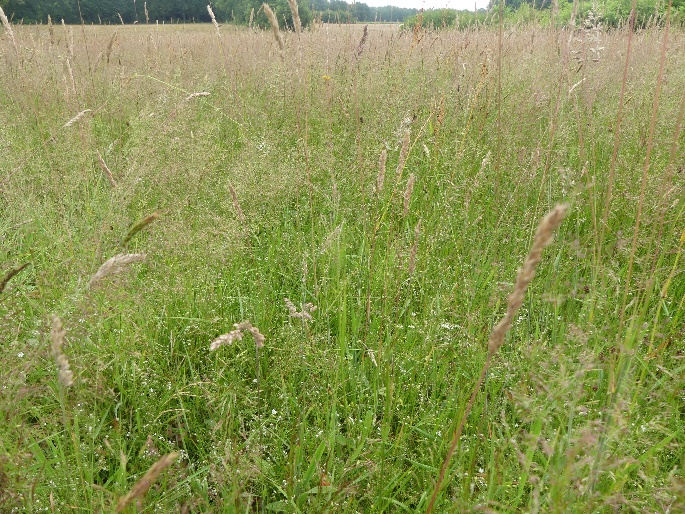 | 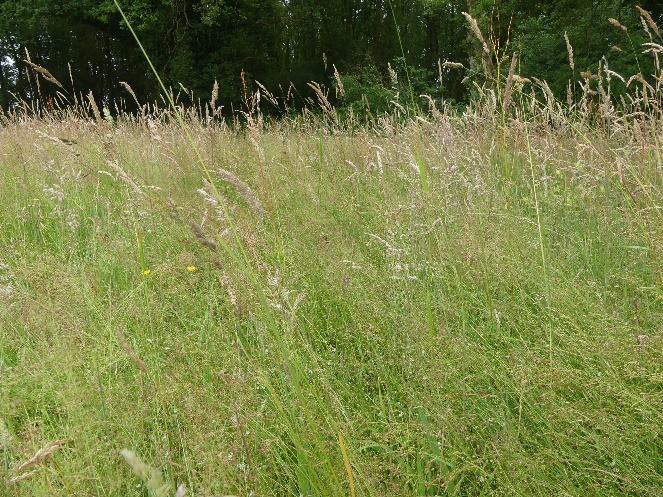 |
|  |  |

Field C pictures on 2 July 2021

| 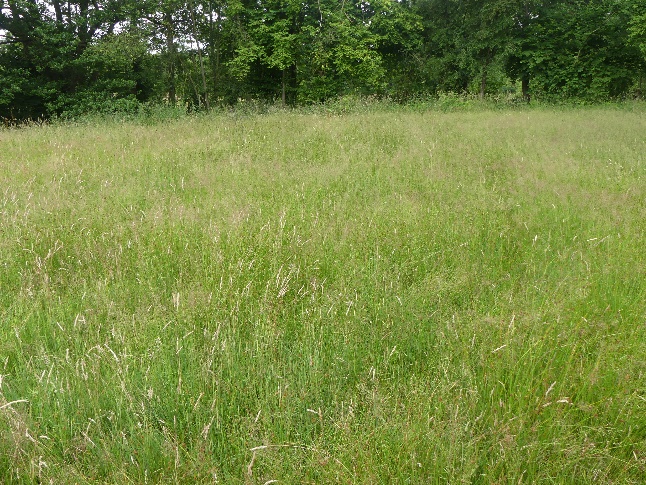 | 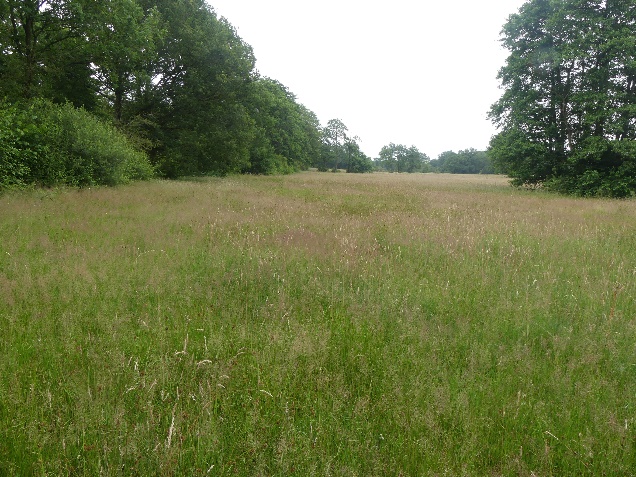 |
| --- | --- |
| 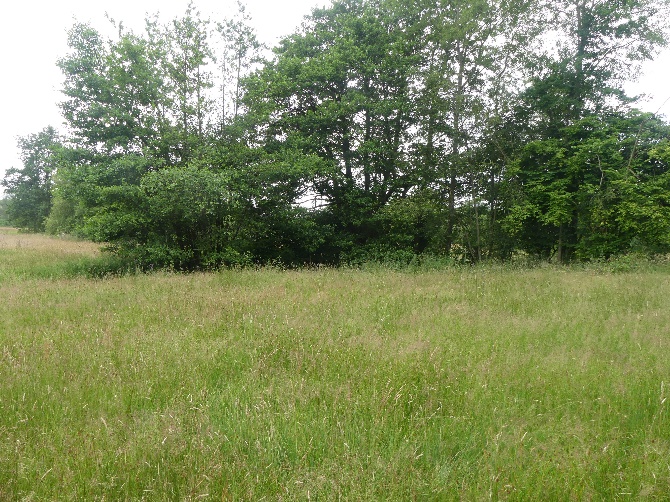 | 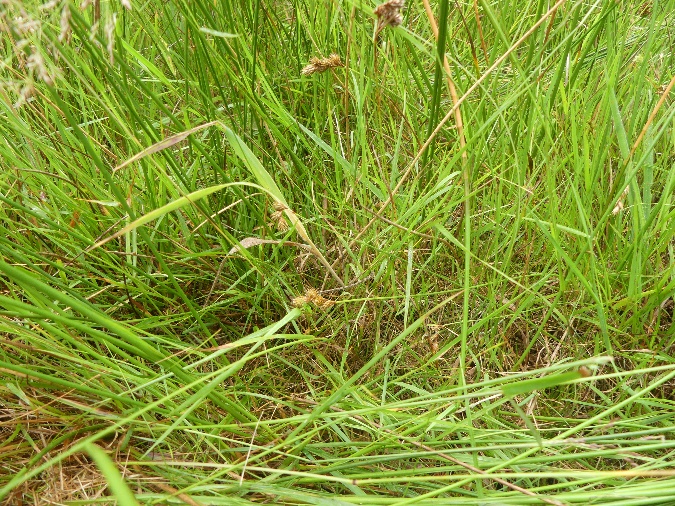 |

Field K pictures on 2 July 2021

| 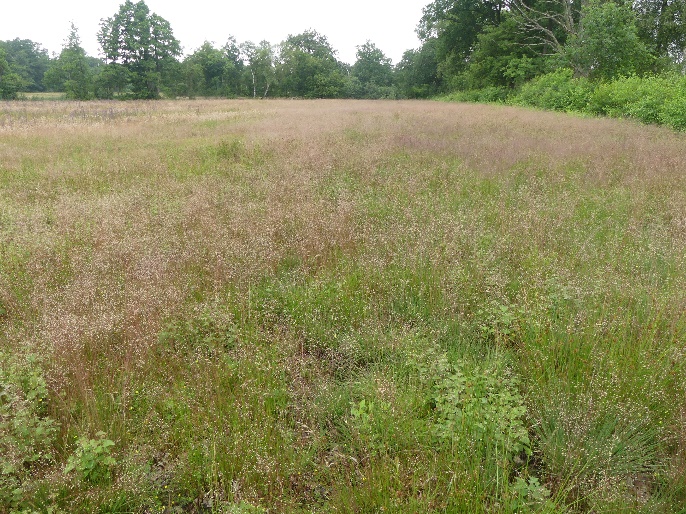 | 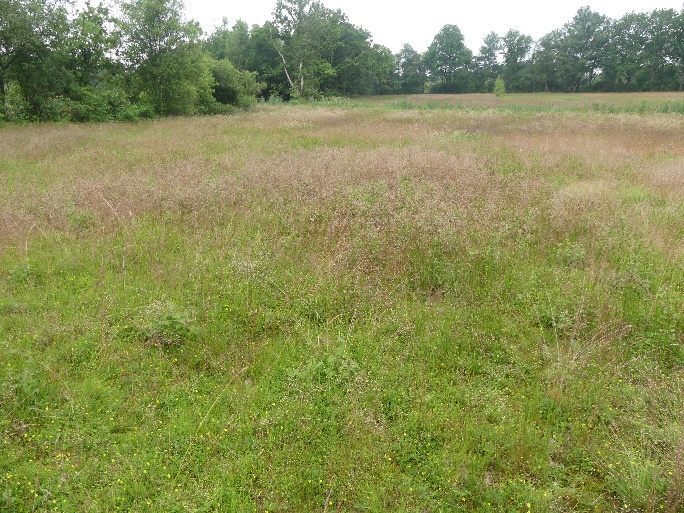 |
| --- | --- |
| 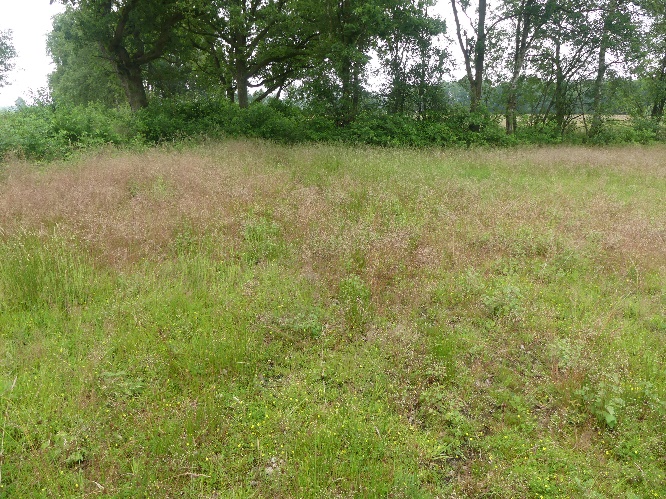 | 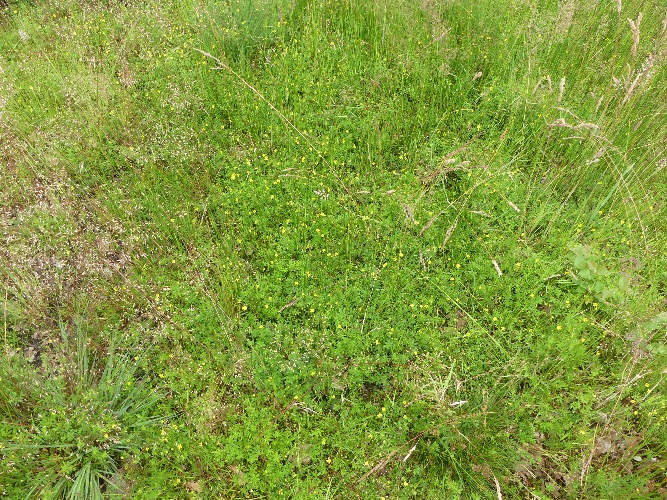 |
|  |  |
